# Rare Extraperitoneal Involvement with Fatal Outcome in a Case of Bilateral Luteinized Thecoma of the Ovaries with Sclerosing Peritonitis

**DOI:** 10.1155/2014/904581

**Published:** 2014-06-05

**Authors:** Mohamed A. Medhat, Mohamed A. Y. Abdel Malek, Saad Zaki, Ahmed Helmy, James J. Driscoll

**Affiliations:** ^1^Department of Tropical Medicine and Gastroenterology, Faculty of Medicine, Assiut University, Assiut 71515, Egypt; ^2^Department of Clinical Pathology, Faculty of Medicine, Assiut University, Assiut 71515, Egypt; ^3^Department of Internal Medicine, College of Medicine, 231 Albert Sabin Way, University of Cincinnati, Cincinnati, OH 45267-0557, USA; ^4^The Vontz Center for Molecular Studies, 3125 Eden Avenue, University of Cincinnati, Cancer Institute, Cincinnati, OH 45267-0508, USA; ^5^The Vontz Center for Molecular Studies, 3125 Eden Avenue, Division of Hematology and Oncology, University of Cincinnati, College of Medicine, Cincinnati, OH 45267-0508, USA

## Abstract

We report the case of a woman diagnosed with bilateral luteinized thecoma of the ovaries with sclerosing peritonitis, multiple intraperitoneal cystic lesions, and extraperitoneal lesions of the liver, inferior to the spleen, and high suspicion of bone marrow involvement. The patient developed profound pancytopenia with rapid clinical deterioration and a fatal outcome.

## 1. Introduction

Thecomas comprise less than 1% of ovarian tumors and are usually diagnosed in postmenopausal women [1]. Less than 10% of the tumors occur in patients less than 30 years of age [2]. Luteinized thecomas (LT) are circumscribed in nests of luteinized appearing cells and were first described by Clement et al. who reported six patients diagnosed with sclerosing peritonitis and LT [3]. They are stromal tumors of the ovary composed of lipid-containing cells resembling those of the theca interna and are essentially benign with the exception of rare mitotically active atypical thecoma [4–6]. LT are also typically estrogen-producing and most commonly occur in women of postmenopausal age (mean age of presentation being 59 and 84% occurring after menopause). The age range of LT patients with sclerosing peritonitis (LTSP) is also broad with cases reported in patients as young as 10 months and as old as 85 years. Clement and colleagues were the first to describe a unique association between LT (or closely related proliferative lesions of the ovary) and SP [3]. In some LTSP cases, sclerosis appears only after the initial oophorectomy [7]. Most LTSP patients present with abdominal pain and/or distention, ascites, pelvic masses, or bowel obstruction. LTSP may also present as secondary amenorrhea [8, 9].

Peritoneal involvement is generally present in LTSP and is readily detected upon macroscopic or microscopic examination. However, the biologic and temporal relationship between the ovarian and peritoneal processes remains incompletely defined. Ovarian biopsies display a characteristic histologic appearance that consists of bland, proliferative spindle-shaped cells along with interspersed clusters of luteinized cells, as well as variable but often marked mitotic activity and frequent marked edema [8]. Release of cells or secretion of a substance from the ovarian lesion that results in fibroblastic or myofibroblastic proliferation have been suggested as putative causes of the associated SP [6]. It has also been speculated that ovarian production of a sex hormone, for example, estrogen or progesterone, is distributed throughout the peritoneal cavity to promote sclerotic lesions. Other substances such as fibrinogenic cytokines, for example, TGF-*β*, are known to play a key role in peritoneal fibrosis [10]. The appropriate treatment for patients with LTSP is also unclear. As it remains uncertain whether the ovaries are secreting or reacting to an offending substance, it is not certain if bilateral salpingo-oophorectomy (BSO) is necessary or beneficial. In several reported cases of LTSP, a clinically uninvolved ovary was left* in situ*, and although some patients had continued complications it was unclear whether their course was significantly worsened compared to those patients in which total oophorectomy had been performed [4]. Suggested treatment includes corticosteroids, GnRH agonists, and antiestrogens [8–10]. Tamoxifen is effective in SP cases that developed after peritoneal dialysis treatment [11]. Prognosis of LTSP patients is different than that of patients with SP due to other causes. The morbidity of patients with LTSP is higher; their clinical course often necessitates relaparotomies, colectomies, and small bowel resection and their course is complicated by fistula formation, short bowel syndrome, and malnutrition [12, 13]. Mortality is variable and depends on the cause of SP [14]. Long-term follow-up and potential metastatic behavior of the ovarian lesion remain uncertain. Extraperitoneal lesions have not been reported, although there have been relatively few cases of LTSP with long-term follow-up and careful monitoring. An extensive study that did follow the course of 20 LTSP patients, over a mean of 5.9 years, did not demonstrate evidence of malignant behavior. As in low-grade malignant sex cord-stromal tumors, it is possible that metastasis could occur very late. Here, we describe a rare, if not unique, case of bilateral LTSP in a woman of child-bearing age with extraperitoneal involvement of multiple organs, highly suspicious for bone marrow (BM) involvement with the presence of pancytopenia and fatal outcome.

## 2. Case Presentation

**Figure 1:**
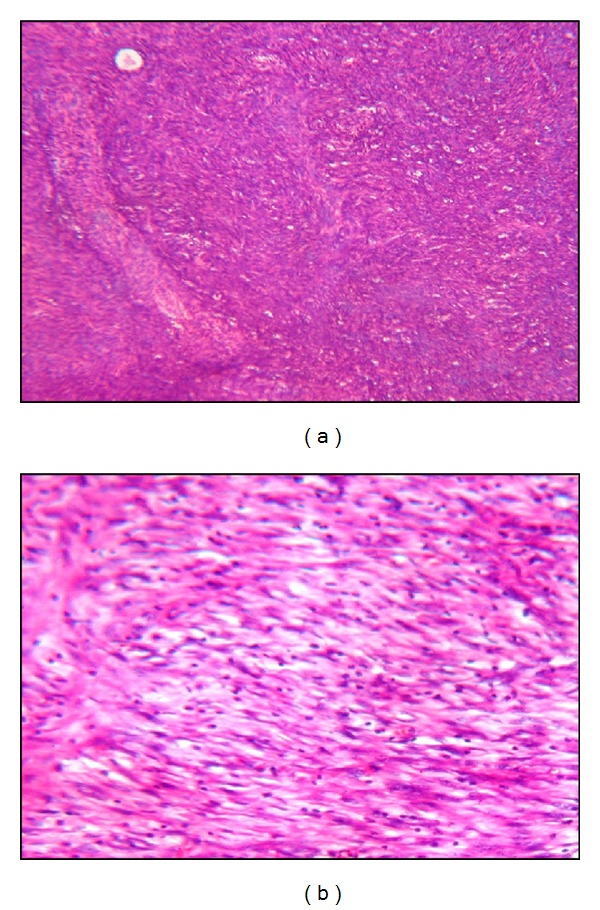
Histology of the ovarian sections. (a) H&E ×100 magnification showing neoplasm made up of proliferated spindled, oval cells with entrapped ovarian follicles. (b) H&E ×200 magnification of the same section in (a).

**Figure 2:**
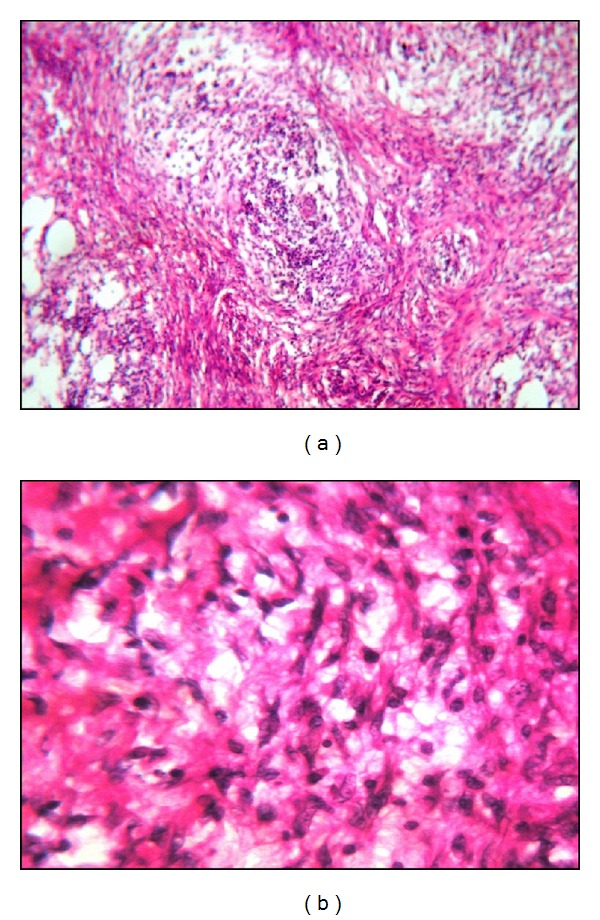
Histology of the omentum. (a) H&E ×100 magnification of omental tissue showing variable sized lobules of fat, separated by fibrocellular bands of reactive fibrosis, infiltrated by acute and chronic inflammatory cells. (b) H&E ×200 magnification. High power view of (a).

A 26-year-old G_1_P_0_ female of Middle-Eastern ancestry was admitted to the intensive care unit at Assiut University Hospital in April 2011 with progressively worsening abdominal distension and repeated vomiting. The patient's gynecologic history included menarche at the age of 13 with subsequent regular monthly menstruation. She had a recent uncomplicated pregnancy with normal weight gain that produced a healthy male child of average weight and length delivered by caesarean section. The child was breastfed for ~20 months with a natural course of weaning as the infant began to eat solid foods. Prior to, during, or after pregnancy the patient did not exhibit any sign of virilization, for example, deepening voice or excess facial hair, on physical examination. Her clinical history began in June 2009 with massive vaginal bleeding that began ~2 months after delivery of her son by caesarean section. At that time, the patient sought medical advice and received a ten-day course of dicynone (ethamsylate) 500 mg, one tablet tid, cyklokapron (tranexamic acid) 500 mg, 1 tablet bid, daflon (diosmin 450 mg and hesperidin 50 mg) 500 mg, 1 tablet tid, and vitamin K (mephyton) 5 mg bid. The bleeding ceased after ten days. Other than lactation-induced amenorrhea, the patient remained well in her usual state of health until December 2010. At that time, she developed the acute onset of diffuse abdominal discomfort associated with abdominal enlargement. She discontinued breast feeding around that time secondary to the deterioration of her overall condition. Abdominal ultrasound revealed marked free ascites with fluid displaying total protein of 35 mg/dL (normal values: 15–45 mg/dL) and a total leukocyte count of 675 cell/mm^3^ with 98% lymphocytes and 2% neutrophils. A preoperative CT scan showed bilateral mixed cystic and enhanced solid lesions involving both ovaries that were free from adjacent structures. The lesions measured 3 × 3 cm (right) and 4 × 3 cm (left) and were associated with marked free ascites and moderate bilateral pleural effusions. The patient underwent subtotal abdominal hysterectomy with BSO, omentectomy, and removal of ~6 L of ascites. Gross pathology indicated a normal-sized uterine corpus in each adnexa and diffusely thickened uterine endometrial lining; both ovaries were irregular and enlarged (right ovary 4 × 3 × 2 cm; left ovary 2.5 × 1.5 × 1 cm). Both ovaries shared an irregular, multinodular outer surface. The cut section was grayish-white to yellow and punctuated by scant small blood-filled cysts. Both fallopian tubes disclosed no significant gross pathology. The omentum showed a large irregular piece of yellow-white hard nodular tissue (16 × 12 cm) that ranged in thickness (1.5 to 2.5 cm). Microscopy of the corpus disclosed grades I-II endometrial hyperplasia without atypia. Sections from both ovaries showed replacement of the ovarian neoplasm with proliferated spindled/oval cells punctuated with variable-sized blood and edema-filled cysts. A few large rounded polyhedral cells with ample cytoplasm (luteinization) were found among the spindled cells. Abnormal mitosis was rather infrequent and a few ovarian follicles were entrapped within tumor cells. No evidence of malignancy was detected (Figure 1). Sections from representative areas of the omentum showed exaggerated variable-sized lobules of fat separated by wide fibrocellular bands of reactive fibrosis infiltrated little acute and chronic inflammatory cells. Again, no evidence of malignancy was detected (Figure 2). Histological examination of the ascites fluid revealed some polymorph nuclear leukocytes and lymphocytes scattered in a hemorrhagic background, also negative for malignancy. The overall clinical picture combined with the biopsy results was consistent with the diagnosis of stage IV LT associated with SP. From January through April 2011, the patient continued to experience diffuse abdominal enlargement and repeated vomiting. Multislice CT of the abdomen and pelvis revealed multiple new peritoneal deposits with diffuse peritoneal thickening and mesentery that coalesced into a precipitating omental cake, moderate ascites, bilateral pleural effusions, no definite pelvic or para-aortic lymph node enlargement, and a mildly enlarged liver without focal lesions. Upper endoscopy revealed antral gastritis treated with a proton pump inhibitor and prokinetic drug.

**Figure 3:**
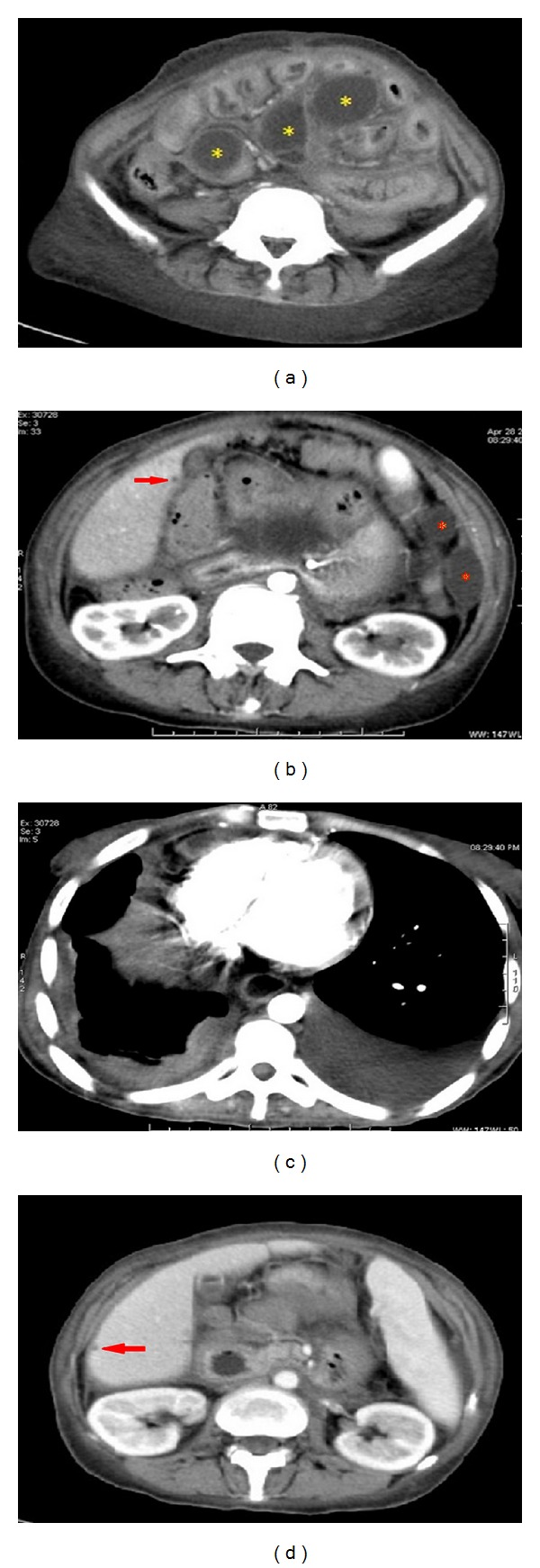
(a) Portal venous phase CT image. Multiple hypodense mesenteric thick walled cystic masses (gold stars) with diffuse intestinal wall thickening. (b) Arterial phase CT image with contrast. Subcentimetric hypodense hepatic focal lesion seen in segment V (red arrows) and two hypodense extraperitoneal cystic lesions seen in left lumbar region (red stars). (c) Bilateral pleural effusions, left side greater than the right. (d) Similar hepatic focal lesion as seen in (b) (red arrow).

**Figure 4:**
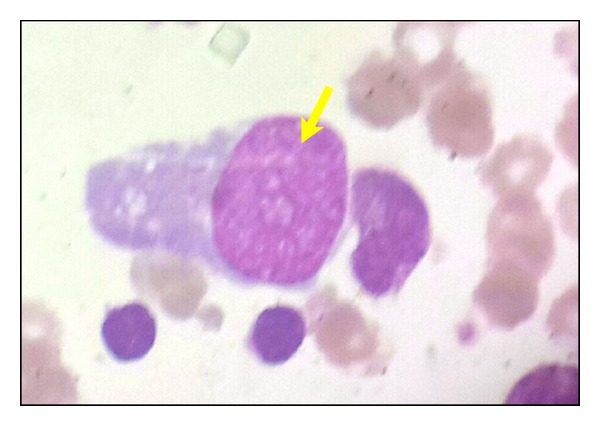
BM film Leishman stained and viewed at ×100 magnification. Shown is a representative field showing an abnormally large mononuclear cell with immature chromatin and pale basophilic cytoplasm (gold arrow) found throughout the aspirate. Based upon morphologic criteria, the abnormally large mononuclear cells appear nonhematopoietic.

Repeated analysis of the ascites and pleural fluid was again negative for malignancy. The patient continued to develop diffuse abdominal wall hardening with recurrence of vomiting. Surgical consultation recommended conservative management that did not improve the symptoms and she required medical admission in April 2011. Physical examination indicated a cachectic, underbuilt woman with pallor, muscle wasting, abdominal hardness, and no sign of virilization. Laboratory values indicated hyponatremia (sodium 128.9 mmol/L), hypokalemia (potassium 2.6 mmol/L), elevated blood urea nitrogen (BUN 10 mmol/L) with normal creatinine, Hb of 103 g/L, and normal leukocyte and platelet counts. Liver function tests were normal apart from a mild increase in the alkaline phosphatase (161 U/L) and low prothrombin concentration (74%). There were no laboratory values to indicate hyperandrogenism or abnormal estrogen or progesterone levels. However, androgen measures have been shown to have a low positive predictive value; therefore, when investigating a patient with rapid onset of virilizing symptoms, the clinical presentation should be used to guide the investigator, even if the androgen profile is normal. Normal androgen levels should not be used as justification for discontinuing the workup in a patient with clinical signs of hyperandrogenism because the best predictor of these neoplasms is the clinical presentation [15]. Follow-up CT showed evidence of multiple intraperitoneal cystic lesions seen insinuating the related small intestinal loops with thickened walls, slight turbid high density contents and septation, measured ~5 × 4 cm with clear regional fat planes, and no infiltration of the adjacent small intestinal loops (Figure 3(a)). There were also two extraperitoneal cystic lesions located in the left lumbar region below the spleen with the same radiological features (Figure 3(b)). The findings were consistent with the presence of cystic intra- and extraperitoneal deposition. Minimal pelvic ascites and bilateral pleural effusions were evident (Figure 3(c)). Haziness of the pelvic mesentery with no residual or recurrent soft tissue masses was seen but mild hepatomegaly and two small hypodense hepatic focal cystic lesions were detected (Figures 3(b) and 3(d)). The patient received enteral feeding and showed improvement but developed low total protein (46 g/L) and albumin (19 g/L) with elevated direct bilirubin (12.2 *μ*mol/L). Hepatitis C antibodies, hepatitis B surface antigen, and HIV antibodies were negative. Complete blood cell count indicated white blood cells (WBCs) of 1.3 × 10^9^/L, red blood cells of 2.1 × 10^12^/L, hemoglobin 55 g/L, platelet count of 7 × 10^9^/L, hematocrit 0.167, MCV 79 fl, MCH 26 pg, and MCHC 328 g/L. The differential count was neutrophils 39%, lymphocytes 54%, monocytes 2%, bands 3%, juvenile 2%, and normoblast 1/100 WBCs. The reticulocyte count was 0.6%, consistent with pancytopenia with leukoerythroblastic reaction. The BM aspirate was hypocellular; all BM elements were depressed and abnormally large scattered mononuclear cells with immature chromatin and pale basophilic cytoplasm were visualized (Figure 4). Further efforts to correct these abnormalities were not successful and the patient passed away three weeks later.

## 3. Discussion

We report the case of a woman of child-bearing age with bilateral LT of the ovaries associated with SP and unique features that include (1) mesenteric, hepatic, and infrasplenic extraperitoneal involvement, (2) evidence to support BM invasion with abnormally large, nonhematopoietic mononuclear cells, and (3) profound pancytopenia with a rapid course and fatal outcome. The precise relationship of these distinct ovarian lesions, often associated with SP and initially considered a variant of LT, remains uncertain. Further extensive immunohistochemical and molecular-genetic studies are needed to better understand the relationship between LT, the extraovarian SP associated with LT, and the extraperitoneal, potentially metastatic lesions described here. Unfortunately, the extensive abdominal hardening and the overall poor clinical status of the patient described here limited the opportunity for successful surgical biopsy of the extraperitoneal lesions to permit further immunohistochemical or molecular studies. In addition, the etiology and temporal relationship between ovarian, extraovarian, and extraperitoneal lesions remains unknown. A theoretical possibility includes metastasis from a primary ovarian lesion and secretion of a substance(s) by the ovarian lesion resulting in a proliferation involving the ovaries and the peritoneum, including the omentum. However, this thinking has been challenged since extraovarian lesions are morphologically bland, mitotically inactive, and not associated with a malignant clinical course [6]. SP may progress even after oophorectomy, as in the patient reported here, to argue against ovarian secretion of activators of fibrosis. However, SP owing to other causes such as practolol therapy or peritoneal dialysis may appear or progress even after withdrawal of the inciting agent, so there is not always an exact temporal relationship of SP with its putative cause [6, 14, 16, 17]. Patients in whom residual ovarian tissue remained* in situ* were somewhat more likely to have continuing complications with peritonitis, but some such patients had no additional problems and several patients had continuing problems long after complete oophorectomy. Therefore, it is difficult to say on clinical grounds whether the ovaries are secreting a fibrosing substance or themselves responding to some stimulus.

The case reported here is highly suspicious for hematologic spread of the primary disease based upon pancytopenia with the leukoerythroblastic reaction in the peripheral blood (PB) accompanied by profound BM hypocellularity, trilineage depression, and presence of abnormal, nonhematopoietic cells. The differential diagnosis for pancytopenia broadly includes aplasia, BM dysplasia, megaloblastic anemia, hypersplenism, leukemias, lymphomas, myeloma, paroxysmal nocturnal hemoglobinuria (PNH), hemophagocytic syndrome myelofibrosis, and infectious processes, for example, tuberculosis or viral. Aplasia was ruled out since there was no history of fever, no infections, and no travel abroad nor occupational, environmental, or radiation exposure. Serology was negative for hepatitis C, hepatitis B, and human immunodeficiency virus. Tuberculosis is excluded as there was an absence of fever, night sweating, lymphadenopathy, leukocytosis, or lymphocytosis. Physical examination did not reveal any lymphadenopathy or manifestations to suggest autoimmune processes. Megaloblastic anemia was excluded since the MCV was within normal limits and there was an absence of erythroid hyperplasia and no evidence of megaloblastic changes in the erythroid or myeloid series. The PB and BM were not consistent with iron deficiency anemia or with combined iron and megaloblastic anemia. In addition, during admission the patient received enteral nutrition supplemented with iron and B12. Hypersplenism was excluded by absence of enlarged spleen on exam and hypocellular BM. Leukemias, lymphomas, myeloma, and myelodysplasia were excluded by absence of characteristic cells for each of these diseases within the PB or BM. Myelofibrosis was excluded based upon the absence of splenomegaly and characteristic teardrop RBCs. PNH was excluded based upon the absence of hematuria and hemoglobinuria, as well as the absence of reticulocytosis, thrombosis, splenomegaly, or erythroid hyperplasia within the BM. Hemophagocytic syndrome was excluded based upon the absence of fever, splenomegaly, and features suggestive of a hemophagocytic syndrome in the BM. Thus, clinical and histopathologic analyses support the relationship between LTSP and BM involvement.

Over the past four decades, numerous reported cases have been diagnosed as thecal tumors of the ovaries, some of which were associated with SP. However, none of the reported cases demonstrated extraperitoneal spread of LTSP. Robert et al. reported 14 cases diagnosed with thecal tumor out of 524 tumors of the ovary seen over a 20-year period [14]. Only one of the cases was diagnosed with histological finding of an LT tumor. Zhang et al. reported 50 ovarian stromal tumors that had a predominant pattern of fibroma or thecoma but also contained cells typical of steroid hormone-secreting cells. 46 tumors were classified as LT and only one case was fatal [5]. However, the cause of death was not classified as LT and there was an absence of clinical or pathological evidence for steroid hormone production at the time of diagnosis. Clement et al. described six patients that had spindle cell proliferations of the ovary resembling LT. Only one fatal case of extensive SP associated with an identical ovarian proliferation was reported. Werness et al. reported 13 cases of LT with SP; however, patient outcome was not reported [10]. Staats et al. reported 27 cases diagnosed with LT of the type typically associated with SP. They reported that LT was fatal in only 3 of 27 cases. The three patients that died were a 76-year-old that died two months after surgery secondary to postoperative pulmonary emboli, a 63-year-old that died secondary to complications from cholecystectomy and related enterocutaneous fistula, and a third patient of 10 months of age that died 4 months after surgery with widespread peritonitis. Hence, it is probable that the cause of death in these three cases was not directly linked to LTSP or extraperitoneal involvement. Bahar et al. reported a fatal case of LT with SP in a 40-year-old woman, who had a history of total abdominal hysterectomy and a left salpingo-oophorectomy. However, the patient died from multiorgan failure approximately 15 months after her diagnostic LTSP surgery [18]. Morizane et al. reported a case of ossifying LT of the ovary in a 51-year-old woman characterized by extensive calcification and metaplastic ossification by histologic exam [19]. However, calcification and ossification would not indicate extraperitoneal spread to or involvement of the BM. From these collective case reports, it is clear that the number of fatal thecoma with SP in young women is extremely low. These findings support our case being the first to report a fatal outcome in LTSP with extraperitoneal lesions and putative BM involvement.

The appropriate and effective treatment for patients with LTSP remains unclear [6]. Since it is uncertain whether the ovaries are secreting or are themselves responding to an offending substance, it is not entirely certain if BSO is necessary in young women that are most frequently affected. Treatment with complete hysterectomy to remove the uterus and oophorectomy or salpingo-oophorectomy is generally curative. Work-up should include abdominal/pelvic CT scan with regular follow-up gynecologic examinations. We recommend a BM biopsy with complete histologic examination to identify potential nonhematopoietic foreign cells. Such cells can then be molecularly and genetically characterized to potentially identify the cell of origin. Future studies are also needed to identify thecoma cells and biomarkers at extraperitoneal sites. New treatment modalities are needed to eliminate thecoma cells within the peritoneum and to prevent the potential spread of thecoma to extraperitoneal sites. Surgical intervention is generally adopted as the primary mode of treatment. A dramatic response to antiestrogens and gonadotropin-releasing hormone agonists with marked symptomatic improvement and complete remission of all abdominal diseases has been reported [6, 16, 17], raising the possibility that these agents may have a role in management. There has been investigation of the use of tamoxifen in idiopathic fibrosing lesions, mainly retroperitoneal fibrosis [20]. A proportion of extraovarian lesions studied were shown to be ER or PR positive, albeit always focally so, in support of the suggestion that hormonal manipulation may play a role in the management of the extraovarian disease, a hypothesis which is worthy of further study [6]. An alternative medical therapy which has been reported is treatment with corticosteroids and several cases in the literature have been treated with hydrocortisone with variable results [21–23]. Again, as most patients improve after surgery without additional therapy, the effectiveness of any therapeutic interventions may be difficult to gauge.

## 4. Conclusion

In conclusion, the ovarian lesions described here exhibit characteristic clinical and pathologic features of LTSP. Similarly, extraovarian sclerosing lesions seen here have been previously described as being consistent with the proliferation of mesothelial or submesothelial cells, but the factor(s) that cause their proliferation remain uncertain [6]. In general, the neoplastic or nonneoplastic nature of the ovarian lesions also remains undefined. Immunohistochemical studies provide no support for a sex cord-stromal neoplasm but also do not exclude it. There are relatively few cases with adequate long-term follow-up and defined extensive immunohistochemical analysis to afford adequate assessment of metastases. As in low grade malignant sex cord-stromal tumors, it is possible that metastasis could occur very late [13]. Therefore, for practical purposes, even mitotically active ovarian lesions of LTSP should be considered clinically benign unless contradicted.

LTSP shares similarities with lymphangioleiomyomatosis (LAM), a rare lung disease that results in the aberrant proliferation of smooth muscle [24]. LAM occurs sporadically to affect only females, usually of child-bearing age, but also occurs in patients of both genders who have tuberous sclerosis. Importantly, the cell of origin in LAM remains to be identified and whether it represents a primary malignancy with metastatic potential or metastasis from another site also is in debate. LAM is associated with inappropriate activation of mammalian target of rapamycin (mTOR), an important intracellular pathway that is also deregulated in cancer cells. As such, sirolimus (rapamycin) has been employed in clinical trials and has shown promise to successfully stabilize lung function in selected LAM patients. LAM cells display several striking similarities to cancer cells including excessive growth, activation of cellular pathways that are also activated in cancer cells, and the hypothesis that LAM cells can metastasize to the lung. However, similar to LTSP, LAM cells under the microscope generally do not appear to be cancerous. LTSP and LAM do not behave like typical cancers. In contrast to cancer, LTSP and LAM does not usually spread to other organs and both pathologies may progress very slowly, over many years [25]. Therefore, although LTSP and LAM both have similarities with cancers, whether these entities should truly be considered as cancer forms remains controversial. Future studies on LTSP with extraperitoneal involvement, and similarly LAM, may determine whether the primary disorder is in fact a cancer and identify cancer-relevant pathways that are deregulated. Chemotherapy would appear to be a reasonable treatment option. In fact, ovarian malignant LT has been shown to be responsive to combination chemotherapy [26]. Future studies may unravel the molecular events responsible for the abnormal growth observed in LTSP, define the cell of origin, and open new lines of investigation that lead to better therapeutic options that improve patient outcome.
